# The evolution of a Na^+^-sensitive *Vibrio cholerae* mutant unmasks the moonlighting aminopeptidase PepA as a regulator of *nhaB* Na^+^/H^+^ antiporter gene expression

**DOI:** 10.1128/jb.00155-26

**Published:** 2026-07-30

**Authors:** Sebastian Herdan, Katharina Kohm, Robert Warneke, Florian Roth, Nadja Glaser, Lucia Villani, Nele Rüping, Lea Dreher, Nicolas Görge, Trung Duc Hoang, Emina Schunke, Claudia C. Häse, Juri Rappsilber, Günter Fritz, Fabian M. Commichau, Johannes Gibhardt, Julia Steuber

**Affiliations:** 1 FG Cellular Microbiology 190 i, Institute for Biology, University of Hohenheim, Stuttgart, Germany; 2 FG Molecular Microbiology 190 h, Institute for Biology, University of Hohenheim, Stuttgart, Germany; 3 FG Bioanalytics, Technical University of Berlin 26524, Berlin, Germany; 4 FG Microbial Processes of Nitrogen Transformation 190 y, Institute for Biology, University of Hohenheim, Stuttgart, Germany; 5 Carlson College of Veterinary Medicine, Oregon State University, Corvallis, Oregon, USA; National Institutes of Health, Bethesda, Maryland, USA

**Keywords:** suppressor mutation, sodium resistance, promoter-up mutation, trigger enzyme, sodium pump, sodium-proton antiporter

## Abstract

The pathogenic bacterium *Vibrio cholerae* is aquatic and can be cultivated in nutrient media with increased salt concentration. To maintain osmotic balance, *V. cholerae* uses Na^+^/H^+^ antiporters and a Na^+^-translocating NADH:quinone oxidoreductase (Na^+^-NQR). The exact contribution of the various Na^+^/H^+^ antiporters to maintaining Na^+^ and H^+^ homeostasis in *V. cholerae* is unclear. However, genetic studies indicate the major Na^+^/H^+^ antiporter NhaA and the Na^+^-NQR are required by the bacteria for Na^+^ resistance under alkaline conditions. Here, we show that the growth defect of a *V. cholerae nhaA nqr* mutant at increased Na^+^ concentrations and alkaline pH is relieved by the rapid acquisition of suppressor mutations. The suppressor mutants could be assigned to two classes. We identified (i) mutations in the promoter of the *nhaB* gene encoding a Na^+^/H^+^ antiporter and (ii) mutations that either affect the expression level of the *pepA* gene or DNA-binding activity of the encoded multifunctional aminopeptidase PepA. The characterization of the *P_nhaB_* and *P_pepA_* promoters of the suppressor mutants, membrane potential measurements, and comparative proteome analyses identified PepA as a novel factor controlling Na^+^ homeostasis in *V. cholerae*.

**IMPORTANCE:**

The emergence and spread of multi-resistant bacteria are a major problem for humans and animals. Therefore, the identification of novel targets for the development of novel antibiotics to combat pathogenic bacteria is extremely important. Since sodium ion homeostasis is an essential process in many pathogenic bacteria, especially those living in marine environments, it represents an interesting target for antibiotics. The perturbation of sodium ion homeostasis does indeed impair the fitness of the human pathogenic bacterium *Vibrio cholerae* but can be restored by the rapid evolution of the bacteria. This suggests that inhibiting multiple targets with different antibiotics might be more effective in preventing the development of resistant bacteria.

## INTRODUCTION

To maintain osmotic balance, all living cells must be capable of extruding sodium ions (Na^+^) from the cytoplasm to the extracellular space. Moreover, bacteria like alkaliphilic *Bacilli* and marine *Vibrio* use Na^+^-driven flagellar motor complexes for locomotion in their aquatic habitats (1). To prevent the accumulation of Na^+^, these bacteria rely on transport systems for the export of the cation. The extrusion of Na^+^ can be achieved with the help of Na^+^/H^+^ antiporters, which are secondary transporters that couple the export of Na^+^ to the import of H^+^ (2–8). For instance, in *Escherichia coli*, the export of Na^+^ is driven by at least two distinct Na^+^/H^+^ antiporters, NhaA and NhaB, of which the former is required for the maintenance of the cytoplasmic pH at alkaline pH (9–13). The expression of the *nhaA* gene is positively controlled by the LysR-type DNA-binding transcription factor NhaR that responds to Na^+^ (14–17). NhaA couples the export of 1 Na^+^ to the import of 2 H^+^ and therefore is crucial for acidification of the cytoplasm under alkaline conditions (18). Moreover, some bacteria can establish an electrochemical sodium gradient using primary Na^+^ pumps, such as the Na^+^-translocating NADH:quinone oxidoreductase (Na^+^-NQR) (19), or the Na^+^-pumping oxaloacetate decarboxylase (20). The Na^+^-NQR is a six-subunit respiratory complex that couples the oxidation of NADH and reduction of ubiquinone to the translocation of Na^+^ across the inner bacterial membrane (21). In *Vibrio cholerae*, the causative agent of cholera disease, the Na^+^-NQR operates together with several Na^+^/H^+^ antiporters (6, 22) to ensure survival in very different habitats, ranging from alkaline marine environments with high salinity to acidic compartments in the human gastrointestinal tract (23, 24). Moreover, virulence and pathogenicity of *V. cholerae* are intimately linked to the activity of the Na^+^-NQR (23, 25), marking the protein complex and other respiratory enzymes of pathogenic bacteria as putative drug targets (26, 27).

In addition to the Na^+^-NQR, five antiporter systems have been identified in *V. cholerae* that contribute to Na^+^ and H^+^ homeostasis (19). For instance, the Na^+^/H^+^ antiporter NhaA in *V. cholerae* (28) is the homolog of NhaA from *E. coli* (29), and the Na^+^-responsive transcriptional activator NhaR from *V. cholerae* complements an *E. coli nhaR* mutant (30). Moreover, NhaB, of which a homolog also exists in *E. coli*, is a Na^+^/H^+^ antiporter that confers resistance to Na^+^ and Li^+^ when overproduced in an *E. coli nhaA nhaB* mutant (6, 31). The K^+^(Na^+^)/H^+^ antiporter NhaP1 also seems to be required for pH homeostasis in *V. cholerae* (32). Furthermore, the multi-subunit Na^+^/H^+^ antiporter Mrp allowed the *E. coli nhaA nhaB* mutant to grow at toxic Na^+^ and Li^+^ concentrations, as well as with natural bile salts (33). While the exact contribution of the Na^+^/H^+^ antiporters NhaB, NhaD, NhaP1, and Mrp in maintaining Na^+^ and H^+^ homeostasis in *V. cholerae* is yet unclear, NhaA can be considered the major Na^+^/H^+^ antiporter that, together with the Na^+^-NQR, was shown to be required by the bacteria for Na^+^ resistance under alkaline conditions (6).

In the present study, we confirmed that mutations in the *nhaA* and *nqr* genes encoding the Na^+^/H^+^ antiporter NhaA and Na^+^-NQR, respectively, are synthetically lethal in *V. cholerae* at high Na^+^ concentrations and pH (6). Serendipitously, we found that the *V. cholerae nhaA nqr* mutant is genomically highly unstable and rapidly forms suppressor mutants in which Na^+^/H^+^ homeostasis is restored. The characterization of the suppressor mutants revealed that restoration of Na^+^/H^+^ homeostasis can be achieved by the strong overexpression of the *nhaB* Na^+^/H^+^ antiporter gene. The suppressor screen also identified the multifunctional PepA protein as a novel repressor of the *nhaB* gene because loss-of-function mutations in *pepA* result in NhaB overproduction and Na^+^ resistance. As an aminopeptidase, PepA is involved in amino acid metabolism, and the protein’s DNA-binding activity controls plasmid segregation and gene expression. The role of PepA in maintaining Na^+^/H^+^ homeostasis in *V. cholerae* is discussed.

## RESULTS

### Genomic stability of a *V. cholerae nqr nhaA* mutant at high salinity and alkaline pH

**Fig 1:**
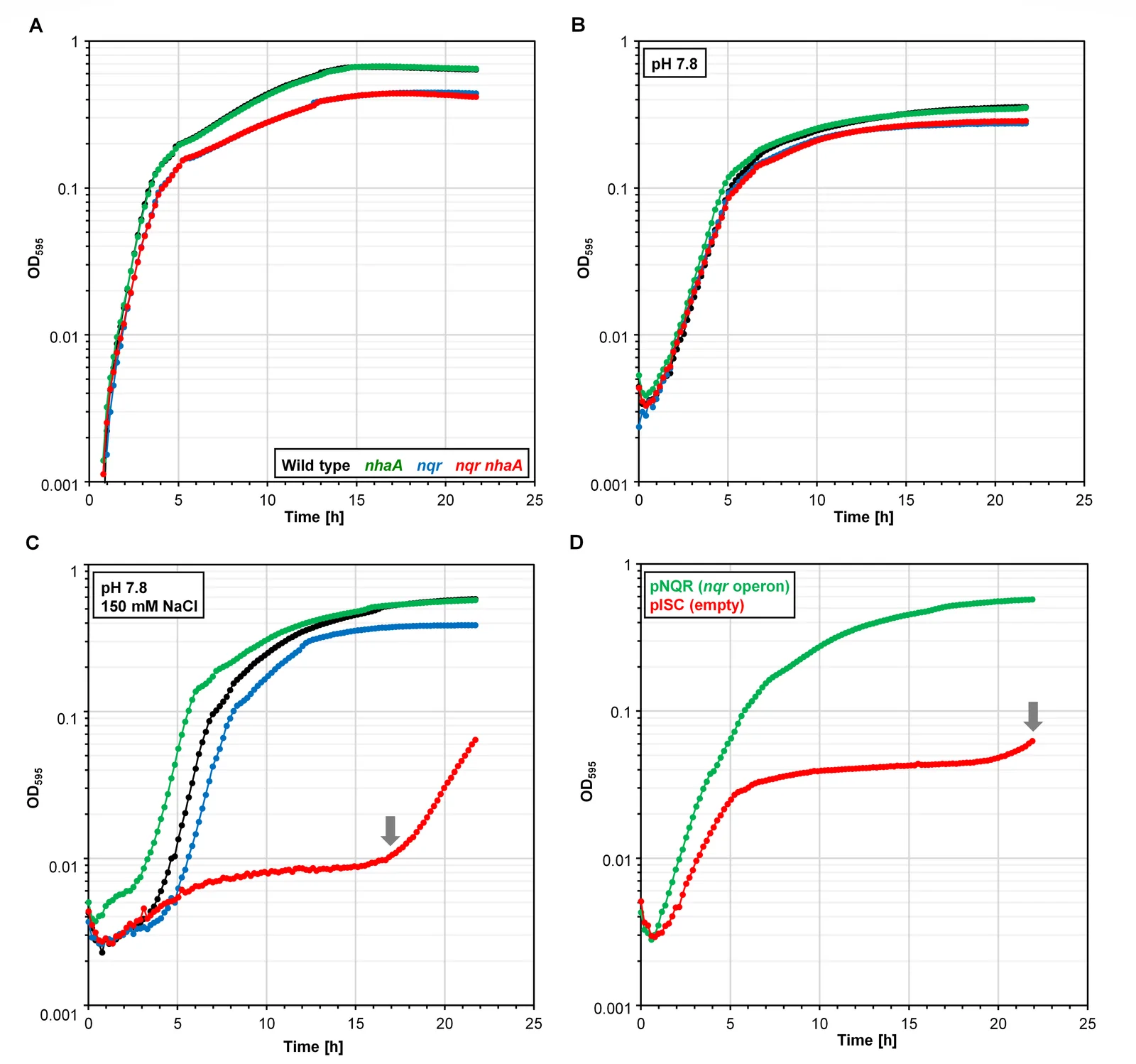
Influence of salinity and pH on growth of *V. cholerae* mutants with impaired Na^+^ export. Growth of the wild type and the *nhaA*, *nqr,* and *nqr nhaA* mutants in LB medium (pH 6.2, 171 mM NaCl) (**A**), in LB_Tris_ (pH 7.8, 13 mM Na^+^) (**B**), and in LB_Tris_ (pH 7.8) in the presence of 150 mM NaCl (**C**). Growth of the *nqr nhaA* mutant carrying the empty plasmid pISC or the plasmid pNQR for the overexpression of the NQR in LB_Tris_ medium at pH 7.8 in the presence of 150 mM NaCl (**D**). The cultivation experiments were carried out in a multi-well plate reader at 30°C. The gray arrows indicate the emergence of suppressor mutants.

Previously, it was observed that the mutational inactivation of the *nhaA*, *nhaB,* and *nhaD* Na^+^/H^+^ antiporter genes in *V. cholerae* did not affect exponential growth of the bacteria (6). Moreover, a *V. cholerae nqr* mutant lacking the NQR complex did not show defects in Na^+^-pumping-related phenotypes (34). This suggests that other secondary Na^+^ pumps can compensate for the lack of NQR. Indeed, the selective inhibition of the NQR by 2-*n*-nonyl-4-hydroxyquinoline *N*-oxide uncovered that the NhaA Na^+^/H^+^ antiporter becomes essential for Na^+^ resistance at alkaline conditions (6). Since the NQR is an interesting target for novel antibiotics, we assessed the genomic stability of a *V. cholerae* strain lacking the major Na^+^/H^+^ antiporter and the primary pump. For this purpose, we cultivated the wild type, the *nhaA* and *nqr* single mutants, and the *nhaA nqr* double mutant in standard lysogeny broth (LB) medium (pH 6.2, 171 mM NaCl) or in LB with Tris-buffered pH (pH 7.8) without additional NaCl (residual Na^+^ concentration, 13 mM). As shown in Fig. 1A and B, the strains lacking the NQR produced slightly less biomass during the stationary growth phase. This indicates some contribution to Na^+^ extrusion by the primary Na^+^ pump under these conditions. In contrast, the *nhaA nqr* mutant showed a severe growth defect in alkaline LB medium containing 150 mM NaCl (Fig. 1C). As expected, the growth defect of the *nhaA nqr* mutant can be relieved by plasmid-based overexpression of the *nqr* operon (Fig. 1D). Thus, in perfect agreement with earlier studies, the *nhaA* and *nqr* genes are synthetically lethal in *V. cholerae* at high salinity and alkaline pH (6, 35). However, after 17 h of growth, a transition to almost wild-type growth rate was observed for the *nhaA nqr* mutant (Fig. 1C). This indicates that this mutant is genomically unstable, and the resulting selective pressure leads to the rapid emergence of suppressor mutations that confer Na^+^ resistance.

### Characterization of *V. cholerae nqr nhaA* suppressor mutants

**Fig 2:**
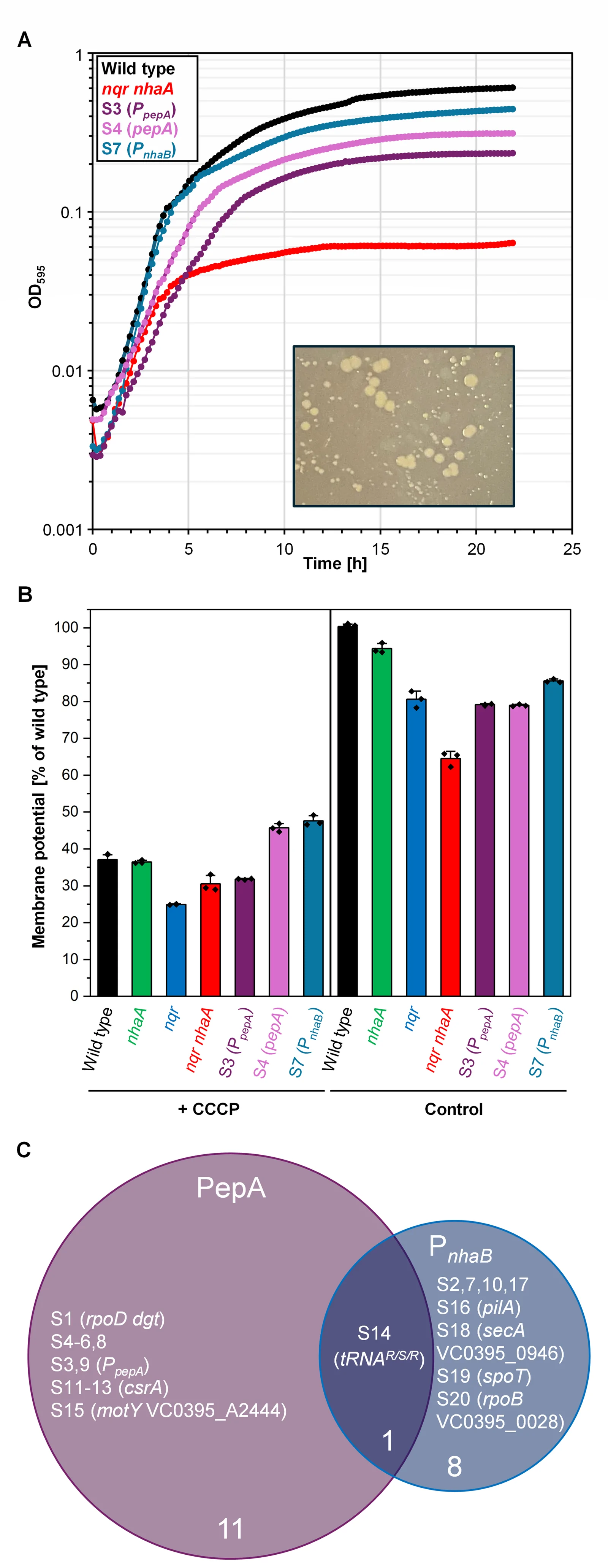
Characterization of selected suppressor mutants and identified genomic alterations relieving the growth defect of the *nqr nhaA* mutant. (**A**) Growth of the wild type, the *nhaA*, *nqr* mutant, and the suppressor mutants S3, S4, and S7 in LB medium at pH 7.8 in the presence of 150 mM NaCl. The cultivation experiments were carried out in a multi-well plate reader at 30°C. The figure inlay shows the suppressor mutants that emerged during growth under selection. (**B**) Determination of the membrane potential in the wild type, the *nhaA,* and *nqr* single mutants, the *nhaA nqr* double mutant, and the suppressor mutants S3, S4, and S7. CCCP, carbonyl cyanide 3-chlorophenylhydrazone. (**C**) Venn diagram illustrating the overlap of the genomic regions mutated in the *nqr nhaA* strain during growth under selection (see also Table 1).

We repeatedly observed that the *V. cholerae nqr nhaA* mutant resumed growth when grown in LB medium at high salinity and pH 7.8 (Fig. 1C and D; data not shown). As described above, this indicates the formation of Na^+^-resistant suppressor mutants that probably overproduce a Na^+^ extrusion system. To understand the underlying molecular mechanism of Na^+^ homeostasis restoration in the background of the *V. cholerae nqr nhaA* mutant, we isolated suppressor mutants. For this purpose, we grew the mutant overnight in standard LB medium (pH 6.2, 171 mM NaCl), washed the cells twice in standard LB, and propagated the bacteria on selective LB plates (pH 7.8, 150 mM NaCl). After incubation for 5 days at 30°C, approximately 250 suppressor mutants appeared on the plates on which 10^7^ cells had been distributed (Fig. 2A). Next, we isolated 20 independent suppressors (S1–S20) (Table 1) and verified the genetic stability of the mutants by passaging the bacteria on standard LB plates and subsequent selection at high salinity and alkaline pH. The cultivation of a selected set of mutants (S3, S4, and S7) in selective LB medium confirmed their Na^+^ resistance. As shown in Fig. 2A, even though the mutants produced less biomass, their growth rates were comparable to that of the wild-type strain. The determination of the membrane potential (Fig. 2B) revealed that all mutants were less impaired in the formation of membrane potential than the parental *V. cholerae nqr nhaA* strain. Furthermore, potentials of the mutants were at least as high as those of the *V. cholerae nqr* strain, thus resembling rescue from the *nhaA* deletion in the parental strain. To conclude, the *V. cholerae nqr nhaA* mutant is genetically unstable and quickly adapts to high salinity and alkaline pH through the accumulation of beneficial mutations.

**TABLE 1:** Isolated *V. cholerae nqr nhaA* suppressor mutants

Suppressor	Genotype^*a*^	Effect of the mutation(s)
S1	*pepA* (G1303T) *rpoD* (C1268T) *dgt* (C686A)	PepA (D435Y) RpoD (A423V) Dgt (S229Y)
S2	*P_nhaB_* (−359T^Ins^)	Altered *nhaB* expression
S3	*P_pepA_* (−162∆T)	Altered *pepA* expression
S4	*pepA* (G676T)	PepA (∆226–503)
S5	*pepA* (G676T)	PepA (∆226–503)
S6	*pepA* (G676T)	PepA (∆226–503)
S7	*P_nhaB_* (G-189A)	Altered *nhaB* expression
S8	*pepA* (T470A)	PepA (∆157–503)
S9	*P_pepA_* (−162∆T)	Altered *pepA* expression
S10	*P_nhaB_* (G-189A)	Altered *nhaB* expression
S11	*pepA* (G491T) *csrA* (A31C)	PepA (R164L) CsrA (T11P)
S12	*pepA* (G491T) *csrA* (A31C)	PepA (R164L) CsrA (T11P)
S13	*pepA* (G491T) *csrA* (A31C)	PepA (R164L) CsrA (T11P)
S14	*pepA* (C490A) *P_nhaB_* (∆−388 – −359 (∆AATAAACTTACTCGAAAGAGGTATTTCT)) ∆71878-72267 (tRNA^Arg^ (VC0395_A0077) tRNA^Ser^ (VC0395_A0078) tRNA^Arg^ (VC0395_A0079))	PepA (R164S), altered expression of *nhaB*, (tRNA^Arg^ tRNA^Ser^) decreased?
S15	*pepA* (T482A) VC0395_A2444 (134∆A) *motY* (C604A)	PepA (V161E), VC0395_A2444 (V46C P47H ∆48–94), MotY (R202S)
S16	*P_nhaB_* (A-345G) *pilA* (G221A)	Altered *nhaB* expression, PilA (G74D)
S17	*P_nhaB_* (A-345G)	Altered *nhaB* expression
S18	*P_nhaB_* (A-12G) *secA* (∆2233–2262 (∆GAGGTTTATAAAGCCAAAGAGCAAGCGGTT)) VC0395_0946 (1013_1014 ins ACCAGA)	Altered *nhaB* expression*,* SecA (∆E741–K750), VC0395_0946 (338_339 ins PE)
S19	*P_nhaB_* (A-12G) *spoT* (C1176A)	Altered *nhaB* expression*,* SpoT (F392L)
S20	*P_nhaB_* (A-12G) *rpoB* (C2623T) VC0395_0028 (C498T)	Altered *nhaB* expression*,* RpoB (R875C)

^*a*^ The identified mutations are specified in relation to the start codon of the indicated genes.

### Identification of the mutations in the *nqr nhaA* suppressors

**Fig 3:**
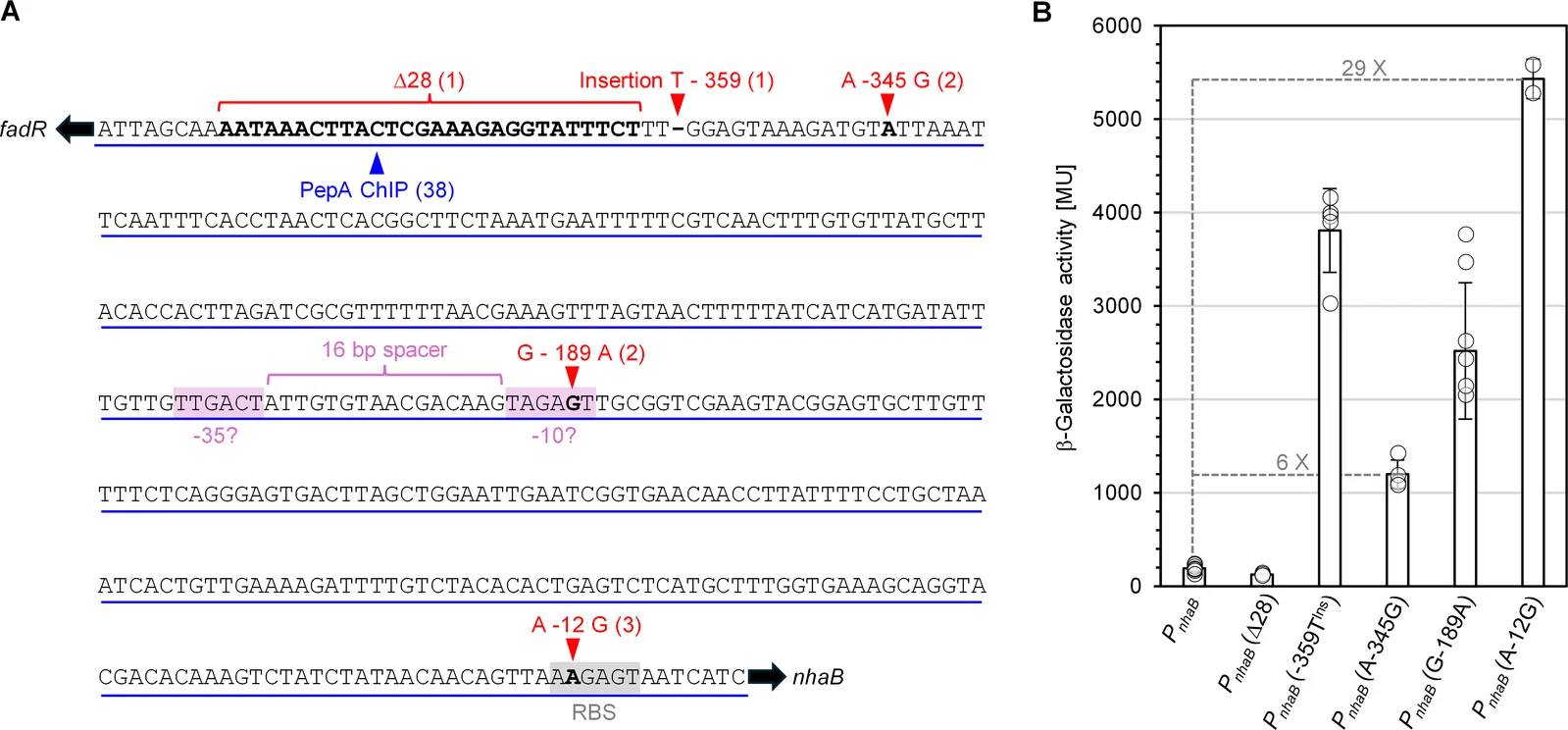
Characterization of the *P_nhaB_* promoter variants of the *nqr nhaA* suppressors. (**A**) *fadR-nhaB* intergenic region located on chromosome 1 of *V. cholerae*. The identified mutations are indicated in red and are specified in relation to the start codon of the *nhaB* gene. The PepA binding site in the *P_nhaB_* promoter is underlined, and the region that was most enriched in the ChIP-Seq experiment (14-fold) is indicated by a blue arrow. RBS, ribosome-binding site. A possible promoter created or improved by the mutation A-189A mutation is highlighted in pink. (**B**) β-Galactosidase assay to assess the effect of the mutations in the *fadR-nhaB* intergenic region on the activity of the *P_nhaB_* promoter in *E. coli* XL1-Blue. The cells were grown in LB medium at 37°C and harvested at an OD_600_ between 0.5 and 0.8. β-Galactosidase activities are given as units per milligram protein. Experiments were carried out at least threefold. Mean values and standard deviations are indicated by columns and error bars.

**Fig 4:**
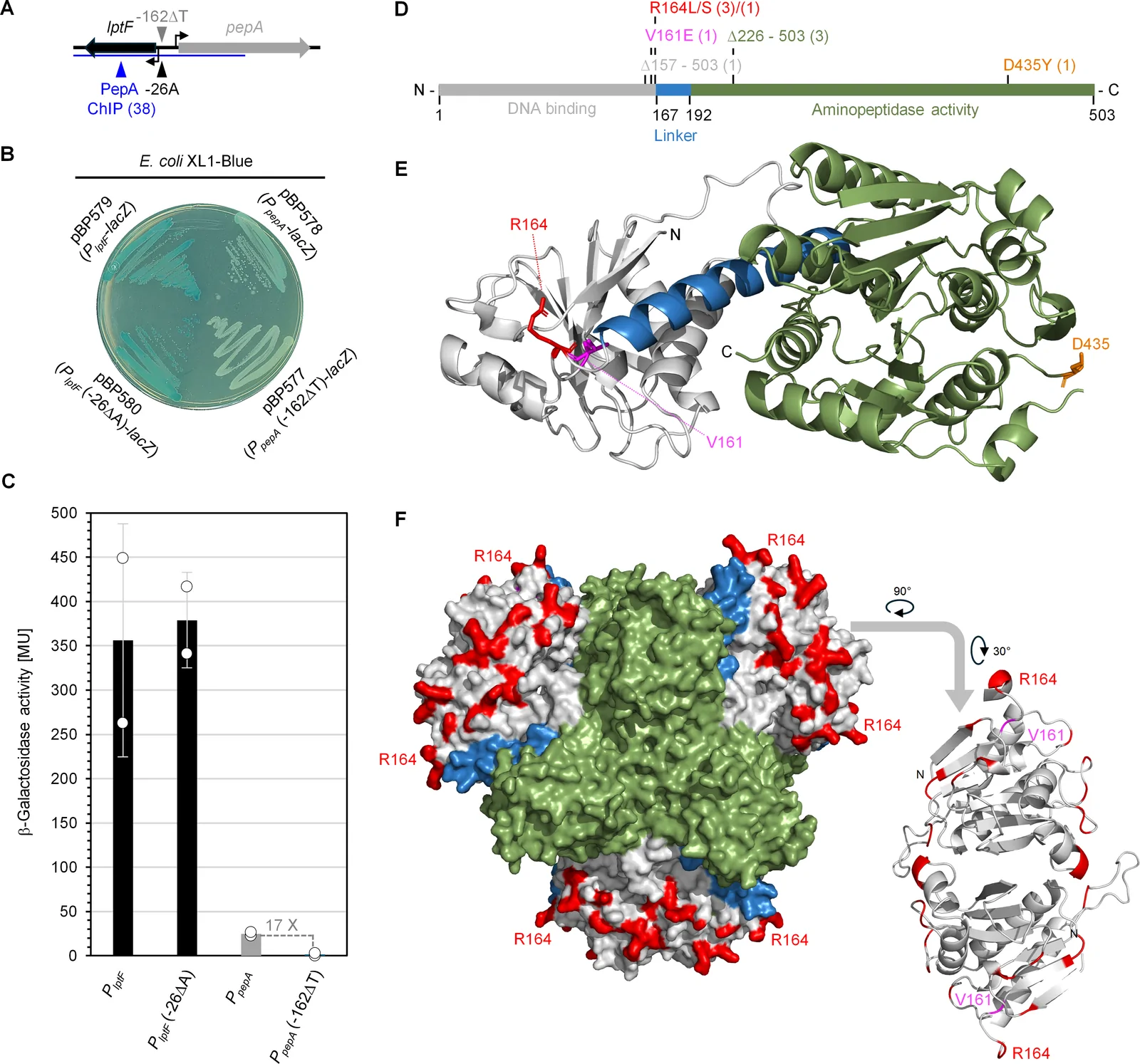
Characterization of the *P_pepA_* promoter variant and PepA variants in the *nqr nhaA* suppressors. (**A**) *lptF-pepA* intergenic region located on chromosome 1 of *V. cholerae*. The identified mutation is specified in relation to the start codons of the genes. The PepA binding site in the *lptF-pepA* intergenic region is underlined, and the region that was most enriched in the ChIP-Seq experiment is indicated by a blue arrow. (**B**) Qualitative test to visualize the activities of the *P_pepA_* and *P_lptF_* promoters in *E. coli* XL1-Blue that was propagated on LB medium containing the chromogenic substrate X-Gal. The plates were incubated for 24 h at 37°C. (**C**) β-Galactosidase assay to assess the effect of the mutation in the *lptF-pepA* intergenic region on the activity of the *P_pepA_* and *P_lptF_* promoters in *E. coli* XL1-Blue. The cells were grown in LB medium at 37°C and harvested at an OD_600_ between 0.5 and 0.8. β-Galactosidase activities are given as units per milligram protein. Experiments were carried out at least threefold. Mean values and standard deviations are indicated by columns and error bars. (**D**) Amino acid replacements in PepA. The numbers in parentheses indicate how often the PepA variants were identified. (**E**) Localization of the amino acid exchanges in a full-length PepA model based on the structure of the *E. coli* homolog (PDB ID: 1GYT). (**F**) Molecular surface of the *V. cholerae* PepA hexamer. The amino acid residues that were previously identified to be important for DNA-binding activity and XerCD-dependent recombination are labeled in red.

Genome sequencing of the 20 isolated suppressors allowed us to assign the mutants to two different classes. Nine mutants had acquired a mutation in a genomic region that likely contains the promoters of the *nhaB* and *fadR* genes (Fig. 2C and 3A). The *fadR* gene encodes a DNA-binding transcription factor that is involved in fatty acid degradation and biosynthesis (36, 37). Since NhaB is a Na^+^/H^+^ antiporter, the mutations in the *fadR-nhaB* intergenic region likely increase the activity of the *P_nhaB_* promoter, which results in overproduction of NhaB and thus Na^+^ resistance of the *V. cholerae nqr nhaA* suppressors. Interestingly, three *P_nhaB_* promoter variants emerged independently of each other twice (Fig. 3A) (Table 1). The remaining 12 suppressor mutants all had acquired mutations that either affect the expression levels of the *lptF* and *pepA* genes or the coding sequence of the *pepA* gene (Fig. 2A and 4A ) (Table 1). The suppressor S14 with a mutation in the *pepA* gene also had deleted a 28 bp-long sequence in the *fadR-nhaB* intergenic region. Recently, chromatin immunoprecipitation sequencing (ChIP-Seq) experiments revealed that PepA is a global transcription repressor in *V. cholerae* (38). Interestingly, the *fadR-nhaB* and *lptF-pepA* intergenic regions were also identified as PepA binding sites (Fig. 3A; Fig. S2). This suggests that the mutations in the *fadR-nhaB* and *lptF-pepA* intergenic regions may abolish the PepA-dependent repression of the *P_nhaB_* promoter and affect negative autoregulation and thus the cellular concentration of the repressor.

As with the first class of mutants, two *pepA* mutations were also identified multiple times (Table 1). Moreover, five *pepA* mutants had acquired additional mutations in genes whose coding products are involved in post-transcriptional regulation (CsrA), Na^+^-driven motility (MotY), cell surface structure (PilA), stringent response (SpoT), information processing (Dgt, RpoB, RpoD), protein secretion (SecA), translation (tRNA^Arg^ tRNA^Ser^), and unknown processes (VC0395_0946, VC0395_A2444) (39–42). However, the fact that in six mutants of the second large group of suppressors, only the *pepA* gene or the *lptF-pepA* intergenic region were altered suggests that the encoded protein is involved in the control of Na^+^ extrusion in *V. cholerae*. Interestingly, observed colony sizes (Fig. 2A, inset) correlated in 10 cases with either mutations in the *pepA* gene (small colonies) or mutations in the promoter region of the *nhaB* gene (big colonies). In summary, the link between PepA and Na^+^ resistance in the *nqr nhaA* suppressors appears less obvious at first glance. However, it is often the case that different mutations produce similar phenotypes in suppressor screens (43–45).

### *nhaB* overexpression restores the growth defect of the *nqr nhaA* mutant

To assess whether the mutations in the *fadR-nhaB* intergenic region of the *V. cholerae nqr nhaA* suppressors affect the activity of the *P_nhaB_* promoter, we constructed translational *nhaB-lacZ* fusions and introduced the plasmids into *E. coli* XL1-Blue. Unfortunately, a *lacZ*-based reporter system to assess the activity of the *P_nhaB_* promoter could not be used in our *V. cholerae* strain because it already contains a *lacZ* gene in the *toxT* locus (Table 2). However, since *E. coli* and *V. cholerae* are both γ-proteobacteria, we assumed that the wild-type *P_nhaB_* promoter and the mutant derivatives must also be active in *E. coli*. It has indeed been shown that a *V. cholerae* DNA-binding regulator can replace the functional homolog in *E. coli* (30). Next, the strains were grown in LB medium, and the cells were harvested during mid-exponential growth (OD_600_ 0.5–0.8). Except for the promoter with the 28 bp-long deletion (derived from suppressor S14), all promoters with single nucleotide exchanges were 6- to 29-fold more active than the wild-type *P_nhaB_* promoter (Fig. 3B). In suppressor S14, the mutation in *pepA* likely causes de-repression of the *P_nhaB_* promoter in *V. cholerae* (see below). To conclude, it is reasonable to assume that, due to the mutations in the *fadR-nhaB* intergenic region, the *V. cholerae nqr nhaA* suppressors overproduce the Na^+^/H^+^ antiporter NhaB, which leads to Na^+^ resistance through increased export of the cation.

**TABLE 2:** Oligonucleotides, plasmids, and bacterial strains

Oligonucleotide	Sequence,^*a*^ 5′→ 3′	Purpose
IS95	CATCACAAACAGAATGATGTACCTG	Sequencing of pAC7 derivatives
IS96	GCAACTGTTGGGAAGGGCG	Sequencing of pAC7 derivatives
FMC15	AAAGAATTCGTCGAATGGCACTCATCATACCACTAGTTG	Amplification of the *P_nhaB_* promoter
FMC16	TTTGGATCCATGATGATTACTCTTTAACTGTTGTTATAGATAGACTTTGTG	Amplification of the *P_nhaB_* promoter
FMC23	AAAGAATTCCTAAAAACAGCACGAAGAAGATCGCAAACTGG	Amplification of the *P_pepA_* promoter
FMC24	TTTAGATCTTCCATGCGTACTCCTACATCCTGAAGACAAATAG	Amplification of the *P_pepA_* promoter
FMC25	AAAGAATTCGCGTATCCTCACTCCTGAAGACAAATAG	Amplification of the *P_lptF_* promoter
FMC26	TTTAGATCTTCCTGGCTTTTTATTGTTTCTCGGATCAAATATCTAACAAATAATCAC	Amplification of the *P_lptF_* promoter
FMC53	TTTGGATCCATGATGATTACTCCTTAACTGTTGTTATAGATAGACTTTGTG	Amplification of the *P_nhaB_* promoter from *V. cholerae* S18
FMC56	AAATCTAGACATGATGATTACTCTTTAACTGTTGTTATAGATAGACTTTGTG	Amplification of the *P_nhaB_* promoter
LV15	AAAGAATTCCCCGGGTCTAGAGTCGACCTGCAGGTCAGCAAGGGCGAGGAGCTGTTCACC	Amplification of *mVenus*
LV16	TTTAAGCTTGGATCCAATCAAAAGGAGAGAGACAAAACTCATTACTTGTACAGCTCGTCCATGC CGAGAGTGAT	Amplification of *mVenus*
nhaA fwd	AAGCCTAGCTACACTCTGGAACA	Verification of the *nhaA* deletion
nhaA rev	CCGCGGTGGTTGACCCCATT	Verification of the *nhaA* deletion
Plasmids	Purpose, insert	Reference
pAC7	Empty plasmid, construction of translational *lacZ* fusions	(46)
pISC	Empty plasmid, expression of genes in *V. cholerae*	(21)
pNQR	Empty plasmid, expression of the *nqr* operon in *V. cholerae*	(47)
pET21a-venus	Template for *mVenus NB*	(48)
pBQ200	Empty plasmid, expression of genes in *E. coli* and *V. cholerae*	(49)
pLV25	Empty plasmid, construction of translational *mVenus NB* fusions	This study
pBP570	*P_nhaB_-lacZ*	This study
pBP571	*P_nhaB_* (G-189A)*-lacZ*	This study
pBP575	*P_nhaB_* (−359T^ins^)*-lacZ*	This study
pBP577	*P_pepA_* (−162∆T)*-lacZ*	This study
pBP578	*P_pepA_-lacZ*	This study
pBP579	*P_lptF_* (−26∆A)*-lacZ*	This study
pBP580	*P_lptF_-lacZ*	This study
pBP585	*P_nhaB_* (A-345G)*-lacZ*	This study
pBP586	*P_nhaB_* (A-12G)*-lacZ*	This study
pBP587	*P_nhaB_* (∆28)*-lacZ*	This study
pBP588	*P_nhaB_-mVenus NB*	This study
pBP589	*P_nhaB_* (−359T^ins^)-*mVenus NB*	This study
pBP590	*P_nhaB_* (∆28)*-mVenus NB*	This study
Bacterial strains	Purpose, genotype	Reference
*E. coli*		
MFD*pir*	Host for pWM91 derivatives and conjugation	(50)
SM10λ*pir*	Host for pWM91 derivatives and conjugation	(51)
XL1-Blue	*recA1 endA1 gyrA96 thi-1 hsdR17 supE44 relA1 lac* [F´ *proAB lacl*^q^ *Z∆M15* Tn*10*(*tet*^R^)]	Agilent
DS941	AB1157 *recF lacZ∆M15*	(52)
SDC1	AB1157 *recF lacZ∆M15 pepA::tet^R^*	(52)
*V. cholerae^b^*		
O395-N1	Parental strain, *lacZ sm*^R^	(53)
O395-N1 (TZ)	*lacZ sm*^R^ *toxT*::*lacZ*	(53)
*nqr*	*lacZ sm*^R^ *toxT*::*lacZ ctxA nqr*	(54)
*nhaA*	*lacZ sm*^R^ *toxT*::*lacZ ctxA nhaA*	(35)
*nqr nhaA*	*lacZ sm*^R^ *toxT*::*lacZ ctxA nqr nhaA*	(35)
S1	*lacZ sm*^R^ *toxT*::*lacZ ctxA nqr nhaA pepA* (G1303T) *rpoD* (C1268T) *dgt* (C686A)	This study
S2	*lacZ sm*^R^ *toxT*::*lacZ ctxA nqr nhaA P_nhaB_* (−359T^Ins^)	This study
S3	*lacZ sm*^R^ *toxT*::*lacZ ctxA nqr nhaA P_pepA_* (−162∆T)	This study
S4	*lacZ sm*^R^ *toxT*::*lacZ ctxA nqr nhaA pepA* (G676T)	This study
S5	*lacZ sm*^R^ *toxT*::*lacZ ctxA nqr nhaA pepA* (G676T)	This study
S6	*lacZ sm*^R^ *toxT*::*lacZ ctxA nqr nhaA pepA* (G676T)	This study
S7	*lacZ sm*^R^ *toxT*::*lacZ ctxA nqr nhaA P_nhaB_* (G-189A)	This study
S8	*lacZ sm*^R^ *toxT*::*lacZ ctxA nqr nhaA pepA* (T470A)	This study
S9	*lacZ sm*^R^ *toxT*::*lacZ ctxA nqr nhaA P_pepA_* (−162∆T)	This study
S10	*lacZ sm*^R^ *toxT*::*lacZ ctxA nqr nhaA P_nhaB_* (G-189A)	This study
S11	*lacZ sm*^R^ *toxT*::*lacZ ctxA nqr nhaA pepA* (G491T) *csrA* (A31C)	This study
S12	*lacZ sm*^R^ *toxT*::*lacZ ctxA nqr nhaA pepA* (G491T) *csrA* (A31C)	This study
S13	*lacZ sm*^R^ *toxT*::*lacZ ctxA nqr nhaA pepA* (G491T) *csrA* (A31C)	This study
S14	*lacZ sm*^R^ *toxT*::*lacZ ctxA nqr nhaA pepA* (C490A) *P_nhaB_* (∆−388 – −359 (∆AATAAACTTACTCGAAAGAGGTATTTCT)) ∆71878-72267^*c*^ (tRNA^Arg^ (VC0395_A0077) tRNA^Ser^ (VC0395_A0078) tRNA^Arg^ (VC0395_A0079))	This study
S15	*lacZ sm*^R^ *toxT*::*lacZ ctxA nqr nhaA pepA* (T482A) VC0395_A2444 (134∆A) *motY* (C604A)	This study
S16	*lacZ sm*^R^ *toxT*::*lacZ ctxA nqr nhaA P_nhaB_* (A-345G) *pilA* (G221A)	This study
S17	*lacZ sm*^R^ *toxT*::*lacZ ctxA nqr nhaA P_nhaB_* (A-345G)	This study
S18	*lacZ sm*^R^ *toxT*::*lacZ ctxA nqr nhaA P_nhaB_* (A-12G) *secA* (∆2233 – 2262 (∆GAGGTTTATAAAGCCAAAGAGCAAGCGGTT)) VC0395_0946 (1013_1014 ins ACCAGA)	This study
S19	*lacZ sm*^R^ *toxT*::*lacZ ctxA nqr nhaA P_nhaB_* (A-12G) *spoT* (C1176A)	This study
S20	*lacZ sm*^R^ *toxT*::*lacZ ctxA nqr nhaA P_nhaB_* (A-12G) *rpoB* (C2623T) VC0395_0028 (C498T)	This study

^*a*^ Recognition sites of restriction enzymes are underlined.

^*b*^ The identified mutations are specified in relation to the start codon of the indicated genes.

^*c*^ The numbers indicate the chromosome coordinates.

### A potential link between DNA-binding activity of PepA and *nhaB* gene expression

The remaining 12 *nqr nhaA* suppressor mutants all had acquired mutations that either affect the expression level or the coding sequence of the *pepA* gene (Fig. 2C) (Table 1). In two suppressors (S3 and S9), the same nucleotide was deleted in the *lptF-pepA* intergenic region (Fig. 4A). While *lptF* codes for an essential component of a lipopolysaccharide transport system, the *pepA* aminopeptidase gene is not essential in *V. cholerae* (38, 55). Therefore, it is unlikely that the mutation in the suppressors S3 and S9 would affect *lptF* expression. To assess the wild-type and mutant *P_lptF_* and *P_pepA_* promoters, we constructed translational *lptF*- and *pepA-lacZ* fusions and introduced the plasmids into *E. coli* XL1-Blue. Next, we propagated the transformants on LB plates containing X-Gal. The *E. coli* cells carrying the wild-type and mutated *lptF-lacZ* fusions both formed dark blue colonies. The β-galactosidase assay confirmed that the mutation in the *lptF-pepA* intergenic region does not affect the activity of the *P_lptF_* promoter (Fig. 4B and C). In contrast, cells with the mutated *pepA-lacZ* fusion formed white colonies on the plates compared to the cells with the wild-type promoter (Fig. 4B). This made it more difficult to identify positive clones using blue-white screening. The β-galactosidase test revealed that the mutated *pepA-lacZ* fusion was about 17-fold less active (Fig. 4C). In summary, the mutation in the *P_pepA_* promoter of the suppressors S3 and S9 likely leads to a reduction of the cellular PepA concentration. Using the *E. coli pepA* wild-type and *pepA::tet^R^* strains, we tested whether *E. coli* PepA has an influence on the *nhaB-lacZ* fusion. However, this was not the case (data not shown).

The remaining 10 suppressors carry mutations in *pepA* that likely affect the DNA-binding activity of the encoded protein (Fig. 2C and 4D) (Table 1). For instance, four suppressors (S4, S5, S6, and S8) had acquired a stop codon that would truncate PepA by 228 or 347 amino acids (Fig. 4B). An extensive characterization of PepA mutant variants from *E. coli* revealed that the N-terminal domain (residues 1–166) is involved in DNA binding, whereas the C-terminal domain (residues 193–503), which is connected by an α-helix (residues 167–192) is required for aminopeptidase activity (Fig. 4D and E) (56, 57). Thus, the truncation in S4–S6 and S8 probably leads to a general defect in PepA folding or oligomerization and thus to the loss of DNA-binding and aminopeptidase activities. The remaining six suppressors contain *pepA* alleles that code for PepA variants with single amino acid exchanges, among them four with replacements of arginine 164 (Fig. 4D and E). The suppressor S15 synthesizes a PepA variant in which a residue located near arginine 164 in PepA was replaced. Arginine 164 was previously identified as crucial for the DNA-binding activity of *E. coli* PepA (56). Therefore, the exchange of arginine 164 and neighboring residues likely affects the DNA-binding activity of *V. cholerae* PepA. The suppressor S1 synthesizes a PepA variant in which aspartate 435 is replaced by tyrosine (Fig. 4D and E). Even though the aminopeptidase domain is altered in this PepA variant, changes in the C-terminal domain can lead to both enzyme functions being impaired (56). As described above, PepA is a global transcription factor that has many chromosomal binding sites, including the *P_nhaB_* and *P_pepA_* promoters (Fig. 3A and 4A) (38). Moreover, suppressor S14 had deleted a part of the PepA binding site in the *P_nhaB_* promoter and synthesizes a PepA variant in which arginine 164 is replaced by leucine (Fig. 3A, 4D and E). Thus, the synthesis of the PepA R164L variant and the deletion of the PepA binding site in the *P_nhaB_* promoter likely have a synergistic effect on *nhaB* expression in *V. cholerae*. In summary, the suppressor analysis suggests that PepA is involved in Na^+^ homeostasis by controlling the expression of the *nhaB* Na^+^/H^+^ antiporter gene.

### Control of *P_nhaB_* promoter activity by PepA

**Fig 5:**
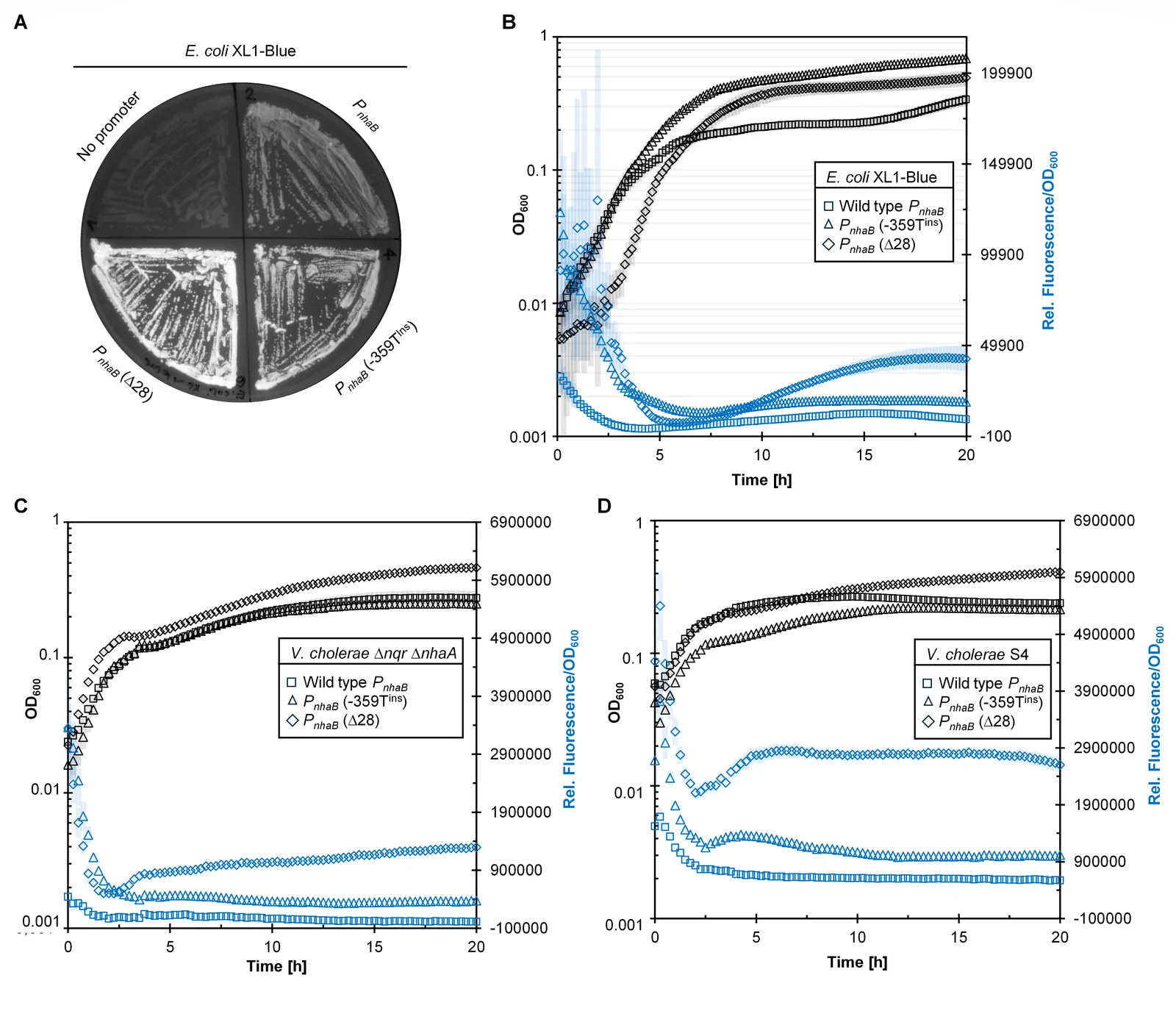
*V. cholerae* PepA controls *V. cholerae P_nhaB_* activity in *E. coli* and in *V. cholerae*. (**A**) Fluorescence of *E. coli* XL1-Blue carrying either pLV25 (empty plasmid, no promoter), pBP588 (*P_nhaB_-mVenus*), pBP589 [*P_nhaB_*(−359T^ins^)-*mVenus*], or pBP590 [*P_nhaB_*(Δ28)-*mVenus*] on LB plates that were incubated for 24 h at 37°C. (**B**) Relative activities of the *P_nhaB_* promoter variants determined by fluorescence signal detection in the *E. coli* XL1-Blue carrying either pBP588 (*P_nhaB_-mVenus*), pBP589 [*P_nhaB_*(−359T^ins^)-*mVenus*], or pBP590 [*P_nhaB_*(Δ28)-*mVenus*] in LB medium. The strain carrying pLV25 (empty plasmid, no promoter) served as control. (**C** and **D**) Relative activities of the *P_nhaB_* promoter variants determined by fluorescence signal detection in the *V. cholerae nqr nhaA* mutant and in suppressor S4 (*pepA* [G676T]), respectively, during growth in LB medium. The relative activity of the *P_nhaB_* promoters is reflected by the mVenus signal divided by the OD_600_.

Next, we tested whether *V. cholerae* PepA regulates the *V. cholerae P_nhaB_* promoter in *E. coli*. For this purpose, we constructed translational P*_nhaB_-mVenus* fusions and introduced the plasmids pLV25 (empty), pBP588 (*P_nhaB_-mVenus*), pBP589 [*P_nhaB_*(−359T^ins^)-*mVenus*], and pBP590 [*P_nhaB_*(Δ28)-*mVenus*] into *E. coli* strain XL1-Blue. When propagated on LB plates, the *E. coli* cells carrying the empty plasmid showed no fluorescence signal (Fig. 5A). In contrast, the cells with the wild-type promoter—and especially those with the two mutant promoter variants *P_nhaB_*(−359T^ins^)- and *P_nhaB_*(Δ28)-*mVenus*—showed strong fluorescence. Cultivation of the strains in LB liquid medium also demonstrated that the mutant promoter variants were much more active than the wild-type promoter (Fig. 5B). The difference in the observed activities of the *P_nhaB_*(Δ28) promoter between the β-galactosidase assay and the mVenus-based assay could be attributed to the fact that the cells for the former assay were harvested during the exponential growth phase (see Fig. 3B). To assess whether PepA controls the activity of the *P_nhaB_* promoter in *V. cholerae*, we transformed the *V. cholerae nqr nhaA* mutant and the suppressor S4 (*pepA* [G676T]) with the plasmids pBP588 (*P_nhaB_-mVenus*), pBP589 [*P_nhaB_*(−359T^ins^)-*mVenus*], and pBP590 [*P_nhaB_*(Δ28)-*mVenus*]. Cultivation of the *V. cholerae nqr nhaA* mutant in LB medium and detection of the fluorescence signal revealed that the *P_nhaB_*(−359T^ins^) and *P_nhaB_*(Δ28) promoters are more active than the *P_nhaB_* wild-type promoter (Fig. 5C). Moreover, all three promoters showed increased activity in the background of the suppressor S4, which carries a mutation in the *pepA* gene. In summary, the promoter-reporter gene analysis supports the hypothesis that PepA directly regulates the expression of the *nhaB* Na^+^/H^+^ antiporter gene in *V. cholerae*.

### Identification of PepA in the *nqr nhaA* suppressors and PepA-regulated genes

**Fig 6:**
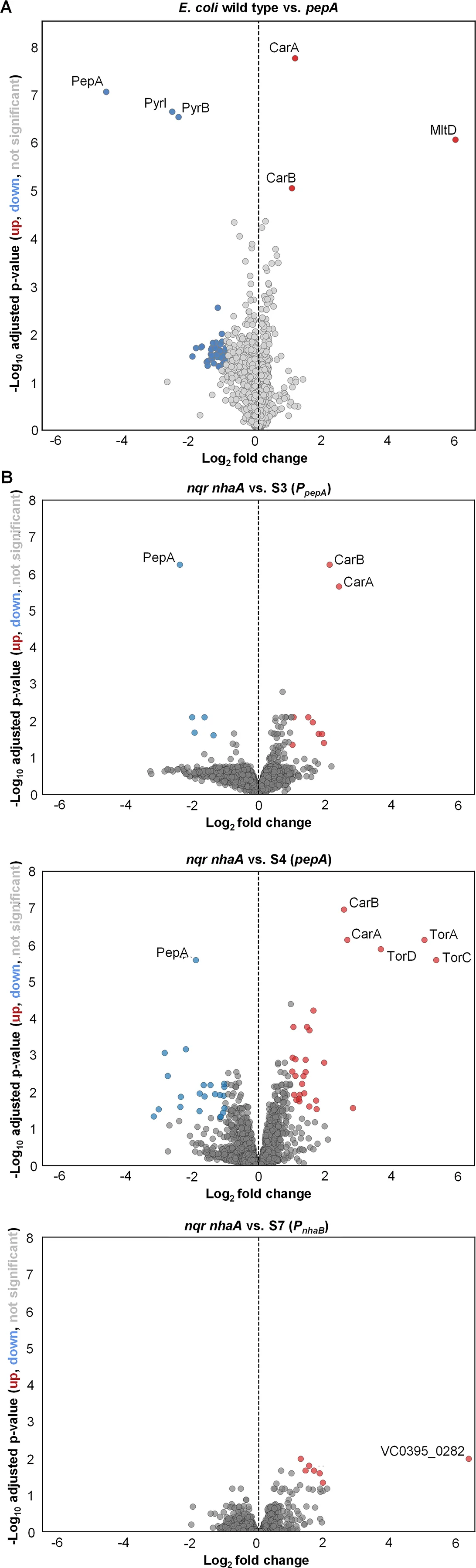
Comparative proteome profiling and chromosomal PepA binding sites. Proteome comparisons of the *E. coli* wild-type (DS941) and *pepA* mutant (SDC1) strains (**A**) and of the *V. cholerae nqr nhaA* mutant and suppressors S3, S4, and S7 (**B**).

To evaluate how the reduced PepA synthesis, the synthesis of a truncated PepA variant, or the increased *P_nhaB_* promoter activity affect the global physiology of the *nqr nhaA* suppressors, we performed comparative proteome analyses. For this purpose, we cultivated the parental strain and the suppressors S3 (*P_pepA_*), S4 (*pepA*), and S7 (*P_nhaB_*) in LB medium under non-selective conditions. As a control, we also compared the proteomes of an *E. coli* strain and its isogenic *pepA* mutant. It is known that PepA represses the *carAB* operon, encodes the CarAB carbamoylphosphate synthetase complex, which is involved in pyrimidine biosynthesis (57–60). Therefore, we expected that there would be more CarA and CarB in *E. coli* Δ*pepA* and possibly also in the *V. cholerae* suppressors S3 and S4. Indeed, both proteins were much more abundant in the *E. coli pepA* mutant and in the two *nqr nhaA* suppressors (Fig. 6A and B). Interestingly, in the *E. coli pepA* mutant, the PyrIB enzyme complex was less abundant compared to the parental strain. PyrIB is also involved in pyrimidine biosynthesis and catalyzes the reaction that follows CarAB. In the *V. cholerae* mutants S3 (*P_pepA_* [−162∆T]) and S4 (*pepA* [G676T]), as expected, PepA abundance was strongly reduced. In contrast, suppressor S7 (*P_nhaB_* [G-189A]; Fig. 6B) showed no changes in PepA abundance. Furthermore, the comparative proteome analyses suggest different metabolic strategies of the suppressor mutants to counteract Na^+^ stress (Fig. 6B; Table S1). For example, in suppressor S7 carrying a mutation in the *nhaB* promoter, which results in increased *nhaB* expression (Fig. 3B), there is a more than twofold increase in levels of citrate lyase subunits CitF and CitC and oxaloacetate decarboxylase subunits OadA-2 and OadG, which are part of the *cit* operon allowing citrate fermentation (61). Moreover, the malate synthase AceB is highly overproduced, likely further facilitating oxaloacetate biosynthesis from isocitrate via the glyoxylate cycle. This will increase overall Na^+^ export capacity with the help of the OadA-2 sodium pump (20), and might decrease carbon flux into the TCA cycle. In contrast, in suppressors S3 (*P_pepA_* [−162∆T]) and S4 (*pepA* [G676T]), in which either expression of the *pepA* promoter is impaired (Fig. 4C) or truncated PepA is expressed, carbon flux through the TCA cycle might be increased. Both the citrate lyase subunits (CitF in S3 and S4, and in S4 also CitD) and OadA-2 (S3 and S4) are less abundant in these mutants, while at the same time there is a moderate increase in citrate synthase subunit GltA in S4. Furthermore, in S4, the fumarase FumC is moderately less abundant, while the succinate-ubiquinone oxidoreductase complex SdhAB is moderately more abundant. Interestingly, in line with the increased abundance of TCA cycle enzymes, enzymes involved in amino acid biosynthetic pathways, especially for glutamate, tryptophan, glutamine, histidine, and others, are also more abundant. Moreover, moderately increased levels of the ATP synthase are observed. This suggests that an increase in membrane potential due to respiratory activity in suppressor S3 and especially S4 facilitates Na^+^ export by the remaining Na^+^/H^+^ antiporters. This is probably accompanied by changes in the membrane structure in suppressor S4, as indicated by alterations in the protein levels of cell-envelope-related proteins such as NlpC, CpxP, and MinCD (Table S1). However, the shapes of the *V. cholerae* reference strain and of *V. cholerae nqr nhaA* and its suppressor strains did not differ significantly (Fig. S1). Changes in abundance of the Na^+^/H^+^ antiporter NhaB itself could not be identified. This is not surprising because NhaB is an integral membrane protein with low recovery. To conclude, the proteome analyses revealed that the mutation in the *P_pepA_* promoter (S3) and in *pepA* (S4) reduce the cellular amount of PepA. Moreover, PepA also regulates *carAB* expression in *V. cholerae,* and changes in the proteomes indicated different adaptation strategies of the *pepA* and *nhaB* suppressor mutants.

## DISCUSSION

In the present study, we found that the *V. cholerae nqr nhaA* mutant with impaired Na^+^/H^+^ homeostasis is genomically unstable during growth in the presence of elevated Na^+^ concentrations at alkaline pH. Similarly, like the *V. cholerae nqr nhaA* mutant, an *E. coli nhaA nhaB* mutant lacking the two specific Na^+^/H^+^ antiporters rapidly forms suppressor mutants at high salinity (62). The characterization of one of these suppressor mutants revealed that the Na^+^ export capacity was partially restored, and the growth rate was like that of the wild type. Although the genomic alteration allowing the growth of this mutant at elevated Na^+^ concentrations was not identified, it was suggested that, under some conditions, *E. coli* can synthesize a primary Na^+^ pump when the Na^+^/H^+^ antiporters NhaA and NhaB are not present (62). It is tempting to speculate that the *E. coli nhaA nhaB* mutant was relieved by a mutation that would affect the synthesis of the other known Na^+^/H^+^ antiporters, like ChaA, MdfA, and MdtM (63–65). However, in contrast to the Na^+^-sensitive *E. coli nhaA nhaB* mutant, in *V. cholerae*, the overproduction of the Na^+^/H^+^ antiporter NhaB can be achieved either by overexpression of the *nhaB* gene or by mutations that affect the synthesis of PepA, a multifunctional protein that has been extensively studied in *E. coli*.

Compared to *E. coli*, *V. cholerae* operates a plethora of electrogenic and electroneutral cation (Na^+^, K^+^)/H^+^ antiporters (6, 22, 28, 31, 33), allowing the organism to tolerate a broad range of salinity and pH in its different ecological niches (24). A complex regulatory network is expected to adjust the cation/H^+^ antiport activities in a given environment, as supported by our study. We previously proposed that the transmembrane voltage generated by NQR is crucial for acidification of the cytoplasm with the help of cation/H^+^ antiporters (66). In this context, it is noteworthy that among the large set of cation/H^+^ antiporters, suppressor mutants identified in this study exhibited upregulation of *nhaB*. With a stoichiometry of 3 H^+^ imported per 2 H^+^ extruded, NhaB seems to be the only system in *V. cholerae* which is capable of achieving acidification of the cytoplasm and export of Na^+^ under the given growth conditions in the Δ*nqr* background.

In *E. coli*, PepA was first identified as an aminopeptidase that allows utilization of peptides as nutrient sources (67). Interestingly, PepA is also a component of a plasmid dimer resolution system that contributes to the segregational and inheritable stability of natural plasmids like ColE1 (68). PepA, together with the DNA-binding regulator of arginine biosynthesis ArgR, serves as an accessory protein, ensuring that the XerCD recombinase-dependent plasmid dimer-to-monomer conversion occurs exclusively unidirectionally (59, 69–76). Recently, it has been observed that PepA might even serve as an RNA-binding chaperone in *E. coli* (77). Moreover, PepA and ArgR are required for the stable maintenance of the P1 prophage and the repression of the *carAB* operon in *E. coli* (57–60, 78). Thus, PepA can be assigned to the class of trigger enzymes, a group of moonlighting proteins that are active in metabolism and in controlling gene expression (79, 80). The regulation of gene expression by trigger enzymes can be achieved by direct binding to DNA or RNA, by modulating the activity of a DNA-binding transcription factor, or by covalent modification of a transcription factor (79, 80). In many cases, the regulatory activity of trigger enzymes is controlled by the presence or absence of a substrate or cofactor. For instance, the aconitase catalyzes the reversible conversion of citrate to isocitrate in the tricarboxylic acid cycle and requires an iron-sulfur cluster for activity (81). Under conditions of iron limitation, the aconitase binds to iron-responsive elements in the mRNAs of genes involved in iron homeostasis (82). However, in contrast to the aconitase, PepA does not require any pyrimidine cofactor to bind to DNA *in vitro* (58). Moreover, the structural characterization and the identification of PepA variants with single and multiple amino acid exchanges revealed that the peptidase activity of the protein is not required for its DNA-binding and XerCD site-specific recombination activity (52, 56–58, 83–85).

**Fig 7:**
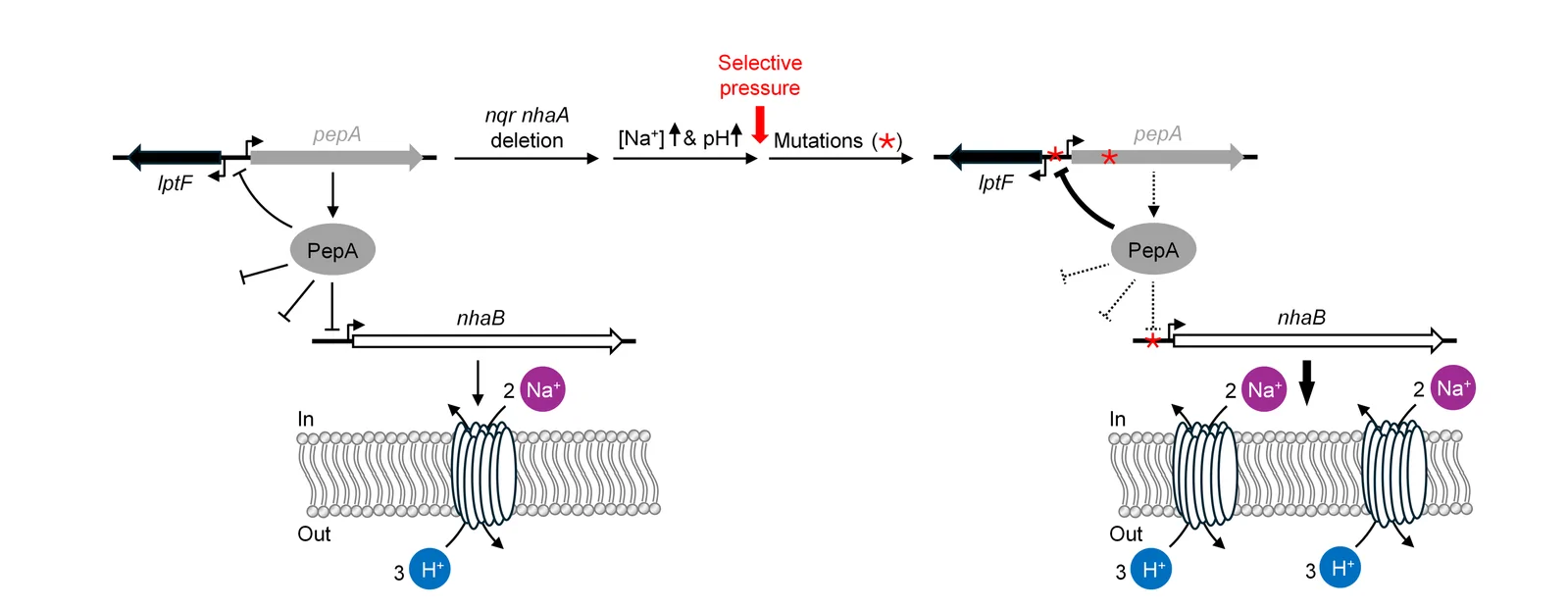
Model for the regulation of the *P_nhaB_* promoter by PepA. The cultivation of the *nhaA nqr* mutant under selection (high salinity and alkaline pH) causes the accumulation of mutations that affect *pepA* expression, the cellular amount of PepA, and *nhaB* expression.

Recently, in *E. coli* and *V. cholerae,* it has been shown that the available carbon source affects the cellular localization of PepA and its DNA-binding activity (38). In the presence of glucose, a carbon source that allows rapid growth, PepA is sequestered to the membrane by a direct interaction with the global transcription regulator CAP (38). During nutrient-limited growth (e.g., growth with acetate as the carbon source), PepA is released from the membrane and becomes active as a DNA-binding transcription factor. It has been hypothesized that the release of PepA from the membrane tailors a stress response to a nutrient-limited environment (38). ChIP-seq analyses identified 293 binding sites for PepA on the two chromosomes of *V. cholerae* O1 biovar El Tor str. N16961, among them the *P_nhaB_* and *P_pepA_* promoter regions (Fig. 3A and 4A; Fig. S2) (38). Our suppressor analysis supports the finding that PepA is a regulator of gene expression and Na^+^/H^+^ homeostasis in *V. cholerae* (Fig. 7). The results of our comparative proteome study obtained with derivatives of *V. cholerae* O395 compare favorably with the findings of Gibson et al. (38), who identified PepA DNA-binding sites in *V. cholerae* N16961. In many cases, PepA binding revealed by ChIP-seq occurred upstream of genes that are also affected in our suppressor strains, as identified by proteomics (Table S1). In other cases, changes in protein abundance are likely needed to redirect the flux of metabolites, for example, toward nitrogen metabolism to prevent bottlenecks or imbalances due to high expression of, for example, CarAB. Some proteins with higher abundance that may directly be regulated by PepA are, for example, the glutamate synthase GltB and the PurDH enzyme complex (moderately increased), which are involved in purine biosynthesis. Their expression likely needs to be coordinated with CarAB to prevent metabolic imbalances. PepA also appears to be involved in the control of toxin gene expression in *V. cholerae* O395 (86). As described above, the DNA-binding activity of PepA is regulated by the available carbon source (38). This indicates that the availability of carbon sources influences the virulence of *V. cholerae*, a phenomenon also observed in many other pathogenic bacteria (87).

As for *V. cholerae,* the PepA binding landscape in *E. coli* was globally mapped by ChIP-seq (38, 88). In contrast to *V. cholerae*, *E. coli* PepA belongs to the group of transcription factors and only binds to two promoters, thereby controlling its own synthesis and the expression of the *carAB* operon (58, 89). The influence of PepA on the cellular concentrations of the PyrIB complex and the membrane-bound lytic murein transglycosylase D (MtlD) is novel and deserves further investigation (Fig. 6A). In *Vibrio anguillarum*, MtlD has been associated with the virulence of the fish pathogen (90). To conclude, PepA is a fascinating protein, and further investigations will be necessary to decipher the significance of this multitasking protein for the global bacterial physiology.

## MATERIALS AND METHODS

### Bacterial strains and chemicals

Bacteria and oligonucleotides (purchased from Sigma-Aldrich, Munich, Germany) used in this study are listed in Table 2. Chemicals and media were purchased from Sigma-Aldrich (Munich, Germany), Carl Roth (Karlsruhe, Germany), and Becton Dickinson (Heidelberg, Germany). Bacterial chromosomal DNA was isolated using the peqGOLD bacterial DNA kit (Peqlab, Erlangen, Germany). PCR products were purified using the PCR purification kit (Qiagen, Germany; New England Biolabs). Phusion DNA polymerase was purchased from Thermo Scientific (Germany) and used according to the manufacturer’s instructions.

### Cultivation of *E. coli* and *V. cholerae*

*E. coli* was grown in LB or in M9 minimal medium at 37°C (91, 92). Agar plates were prepared with 15 g agar/L (Roth, Germany). *E. coli* and *V. cholerae* cells that were transformed with the plasmid pAC7 and its derivatives were selected on LB plates supplemented with 100 µg/mL ampicillin and 80 µg/mL X-Gal. *E. coli* transformed with pBAD33 and its derivative was selected with 30 µg/mL chloramphenicol. *V. cholerae* was grown in LB, LB_sucrose_, LB_Tris_, and LB_Tris_-NaCl medium. LB medium contains 1% tryptone (wt/vol), 0.5% yeast extract (wt/vol), 171 mM NaCl, and has a pH of 6.2. LB_sucrose_ contains 1% tryptone (wt/vol), 0.5% yeast extract (wt/vol), and 10% sucrose (wt/vol). LB_Tris_ medium was used to monitor the effect of Na^+^ on the growth and contains 100 mM Tris. The pH was adjusted to 7.6 with HCl at room temperature, which corresponds to pH 7.8 at 30°C. The Na^+^ concentration of this medium was determined to be 13 mM using atomic absorption spectroscopy. LB_Tris_-NaCl medium was supplemented with 150 mM NaCl. *V. cholerae* strains were grown at 30°C and selected with 50 µg/mL streptomycin. Growth of *V. cholerae* strains in liquid medium at 30°C was monitored in 24-well plates (Thermo Scientific, USA) in an Infinite F200 Pro plate reader (Tecan, Switzerland), and the OD_595_ was measured at 10 min intervals for 24 h. For this purpose, the *V. cholerae* strains were cultivated overnight in LB_Tris_ medium at 180 rpm and 30°C. The cultures were normalized to an OD_600_ of 0.8. Next, a 24-well plate was prepared with 1 mL of growth medium in triplicate. After inoculation with 10 µL (1%) of the normalized overnight culture, growth of three biological replicates was monitored with the plate reader.

### Plasmid construction

Plasmids used and constructed in this study are listed in Table 2. Translational promoter-*lacZ* fusions for determining the activities of the *P_nhaB_*, *P_pepA_*, and *P_pepA_* wild-type and mutated promoters were constructed as follows. The *P_nhaB_*, *P_pepA_*, and *P_lptF_* promoters were amplified by PCR using the oligonucleotide pairs FMC15/FMC16, FMC23/FMC24, and FMC25/FMC26, respectively. The primer pair FMC15/FMC49 was used to amplify the *P_nhaB_* promoter variant from suppressor S18. The *P_nhaB_* promoter fragments (615 bp) were digested with *Eco*RI and *Bam*HI and ligated with pAC7 (46), which was cut with the same enzymes. *P_pepA_* and *P_lptF_* (193 bp) promoter fragments were digested with *Eco*RI and *Bgl*II and ligated to the *Eco*RI/*Bam*HI-linearized pAC7 plasmid. The plasmid pLV25 was constructed to monitor the activities of translational promoter-*mVenus NB* fusions by fluorescence measurements. For this purpose, the *mVenus NB* gene was amplified from plasmid pET21a-venus (48) using the primer pair LV15/LV16 and ligated to the plasmid pBQ200 (49), which was cut with *Eco*RI and *Hind*III. The primer pair FMC15/FMC56 was used to amplify the *P_nhaB_* promoter variants [*P_nhaB_*, *P_nhaB_*(−359T^ins^), and *P_nhaB_*(Δ28)], which were introduced into the *Eco*RI/*Xba*I-digested plasmid pLV25. The resulting plasmids pBP588 (*P_nhaB_-mVenus*), pBP589 [*P_nhaB_*(−359T^ins^)-*mVenus*], and pBP590 [*P_nhaB_*(Δ28)-*mVenus*] were used to assess the *P_nhaB_* promoter activity in *E. coli* and in *V. cholerae*. All plasmids were verified by Sanger sequencing (Microsynth-SeqLab Sequence Laboratories, Göttingen, Germany).

### Construction of *V. cholerae* strains

The nontoxic variant of the *V. cholerae* Ogawa 395 strain (O395-N1), denoted “wild type” (53, 93), and the *nqr* mutant, a derivative of the strain O395-N1 lacking the *nqrABCDEF* operon (54), were used as parental strains (Table 2). The plasmid pWM91 *nhaA* was isolated from the *E. coli* strain SM10λ*pir* and used for transformation of the *E. coli* strain MFD*pir*. To obtain a chromosomal deletion of the *nhaA* gene in *V. cholerae*, plasmid pWM91 *nhaA* was transferred to the *V. cholerae* wild type and *nqr* mutant by conjugation using the *E. coli* donor strain MFD*pir*-pWM91 *nhaA*. The *V. cholerae nhaA* and *V. cholerae nqr nhaA* mutants were selected on LB_sucrose_ medium, and deletion of the *nhaA* gene was confirmed by colony PCR with the oligonucleotide pair nhaA fwd/nhaA rev. *V. cholerae* strains were transformed with plasmid DNA as previously described (94).

### Membrane potential measurements

Membrane potential of *V. cholerae* cells was measured using the BacLight bacterial membrane potential kit (Invitrogen) as described before (66). Briefly, bacteria were grown in LB_Tris_-NaCl (30°C, 180 rpm shaking) for 4 h, harvested (20,000 × *g*, 5 min, room temperature), washed in PBS (137 mM NaCl, 2.7 mM KCl, 10 mM Na_2_HPO_4_, 1.8 mM K_2_HPO_4_, pH 7.4), and adjusted to an OD_600_ of 0.25. Depolarization control was achieved by addition of 5 µM CCCP (carbonyl cyanide 3-chlorophenylhydrazone) and incubation for 15 min in the dark at 22°C. Cell suspensions without DiOC_2_ (3,3′-diethyloxa-carbocyanine iodide) and with DMSO served as controls. Staining was performed with 30 µM of DiOC_2_. Samples (200 µL) from three different cultures per strain were measured in black-bottomed 96-well plates (polystyrene, 4titude) in a Tecan Infinite F200 Pro plate reader (excitation: 485 nm/20 nm; emission: 535 nm/25 nm, 635 nm/35 nm). Background fluorescence of cell suspensions without DiOC_2_ was subtracted. Fluorescence intensities were normalized to that obtained with the wild-type strain.

### Genome sequencing and plotting of PepA binding sites

Genomic DNA was prepared from 2 mL overnight cultures (LB_Tris_-NaCl) using the peqGOLD Bacterial DNA Kit (VWR, USA) following the instruction manual. Purified genomic DNA was paired-end sequenced (2 × 150 bp) (Azenta, Leipzig, Germany). Sequencing reads were mapped to the *V. cholerae* genome (chromosome 1, CP000626.1; chromosome 2, CP000627.1) using the Geneious (v.2025.2.2, Docmatics) software package (95). Genomic regions of PepA binding sites as identified in ChIP experiments (38) of *V. cholerae* O1 biovar El Tor str. N16961 (NC_002505.1 for chromosome 1 and NC_002506.1 for chromosome 2) were extracted and mapped to the genome of *V. cholerae* O395-N1 (CP000627 for chromosome 1 and CP000626 for chromosome 2) using the Geneious (v.2025.2.2, Docmatics) software package (89% similarity [cost matrix 93% similarity (5.0/−9.026168) and “only transfer best match” settings] (95).

### β-Galactosidase assay

Quantitative studies of *lacZ* expression in *E. coli* were performed as follows: cells were grown at 37°C in LB medium. Cells were harvested at an optical density (OD_600_) of 0.6 to 0.8. Specific β-galactosidase activities were determined with cell extracts obtained by lysozyme treatment as described previously (96). One unit of β-galactosidase is defined as the amount of enzyme that produces 1 nmol of *o*-nitrophenol per minute at 28°C. The Bio-Rad dye-binding assay was used to determine the protein concentrations.

### Monitoring *P_nhaB_* promoter activity in growing *E. coli* and *V. cholerae* cells

To assess expression of the different P*_nhaB_*-*mVenus* constructs (pLV25 [empty], pBP588 [P*_nhaB_*], pBP589 [P*_nhaB_* (−359T^ins^]), and pBP590 (P*_nhaB_* [Δ28]), *E. coli* XL1-Blue cells harboring the plasmids were streaked on LB ampicillin plates (100 µg/mL) from overnight cultures and incubated overnight at 37°C. Plates were subsequently imaged using the UVP ChemStudio (Analytik Jena) with blue light excitation (λ^ex^ 445 nm–470 nm) and the green filter set (λ^em^ 513 nm–557 nm) for 50 ms.

To assess the expression dynamics, the same *E. coli* cells were cultivated in liquid medium with ampicillin (100 µg/mL) at 160 rpm and 37°C. LB overnight cultures were used to inoculate fresh LB medium to an OD_600_ of 0.1. These cultures were used to inoculate 96-well plates (Sarstedt) with the same medium to an OD_600_ of 0.1. Growth and mVenus fluorescence of at least three biological replicates were monitored using a BioTek Synergy H1 microplate reader (Agilent) at 37°C and double orbital shaking at 282 cpm (3 mm). The OD_600_ and mVenus fluorescence (λ^ex^ 509 ± 10 nm; λ^em^ 535 ± 15 nm; gain 65; bottom optics) were measured every 10 min. Results were analyzed using the Gen5 software (v.3.16). *V. cholerae* cells were cultivated in the presence of 200 µg/mL ampicillin at 180 rpm and 37°C in LB medium. Overnight cultures in LB medium were used to inoculate fresh LB medium to an OD_600_ of 0.1. These cultures were used to inoculate 96-well plates (Sarstedt) with the same medium to an OD_600_ of 0.1. Growth and mVenus fluorescence of at least three biological replicates were monitored using a SpectraMax iD5e microplate reader (Molecular Devices) at 37°C and double orbital shaking at 100 rpm (1 mm). The OD_600_ and mVenus fluorescence (λ^ex^ 500 nm; λ^em^ 540 nm; medium gain; bottom optics) were measured every 15 min. Results were analyzed using Microsoft Excel and Origin(Pro), version 2025, OriginLab Corporation, Northampton, MA, USA.

### Electron microscopy

Bacterial cultures were grown at 30°C in LB medium for 4 h. Cells were harvested (13,000 × *g*, 5 min, 4°C) and resuspended in PBS. Samples were adjusted to an OD_600_ of 0.1 and washed twice in PBS. Cells were fixed in fixative solution (50 mM HEPES, pH 7.2, 1% *p*-formaldehyde, 2.5% glutaraldehyde) for at least 2 h. After fixation, samples were gently washed three times in PBS (13,000 × *g*, 5 min, 4°C) and applied on poly-L-lysine-coated glass slides. After stepwise dehydration in an ethanol series (5 min in each of 30%, 50%, 70%, 80%, 90%, and 96% EtOH), samples were critical-point dried in CO_2_ (K850 Critical Point Dryer, Quorum, United Kingdom), coated with 8 nm carbon (Q150T ES plus, Quorum, United Kingdom), and examined with a scanning electron microscope (EVO 15, Everhart-Thornley Detector, Zeiss, Oberkochen, Germany) at 8 kV and a sample current of 150 pA.

### Proteome analyses

Bacterial cultures were grown at 30°C in LB medium for 4 h. Cells were harvested (13,000 × *g*, 5 min, 4°C) and resuspended in PBS. Cell pellets of three biological replicaes es were lysed in 60 µL trifluoroacetic acid at 70°C for 3 min, before 600 µL of 2 M Tris Base was added. The cell lysates were cleared by centrifugation for 5 min at 16,000 × *g* at 4°C. Protein concentrations of the cleared lysates were determined by NanoDrop, and 10 µg of the proteins was subjected to tryptic digestion (1:100 for 18 h at 37°C). The peptides were desalted on Evotip using the standard producer protocol and washed twice with 100 µL solvent A (0.1% TFA (vol/vol) in water). Peptides were eluted twice in solvent B (80% ACN, 0.1% TFA [vol/vol] in water) and vacuum dried. The samples were resuspended in 20 μL 0.1% (vol/vol) formic acid, 1.6% (vol/vol) acetonitrile. For each sample, 0.4 μL injections were performed. The peptides were separated on a 50 cm μPAC Neo HPLC Column (Thermo Scientific) operating at 50°C column temperature. Mobile phase A consisted of 0.1% (vol/vol) formic acid and mobile phase B of 80% (vol/vol) acetonitrile with 0.1% (vol/vol) formic acid. After loading on the column, peptides were separated first at a flow rate of 750 nL/min for 4.95 min, then 500 nL/min for 3.15 min. Separation was achieved using a linear gradient from 4% to 8% mobile phase B over 0.25 min, then to 20% over 4.7 min, and subsequently to 35% over 2.25 min. The gradient was followed by a rapid increase to 99% mobile phase B within 0.9 min. Eluted peptides were ionized by an EASY-Spray source (Thermo Scientific) and introduced directly into the mass spectrometer. The MS data were acquired in the data-independent (DIA) mode. For precursors with m/z range of 380–980, isolated with 3 m/z isolation windows (0 overlap), resulting in 200 scan events per acquisition cycle. Isolated precursor ions were fragmented using higher-energy collisional dissociation with a normalized collision energy of 25%. MS2 spectra were recorded in the Astral mass analyzer over an m/z range of 150–2,000. In parallel, one MS1 spectrum was acquired every 0.6 min in the Orbitrap at a resolution of 240,000 (at m/z 200) over an m/z range of 380–980.

### Processing of mass spectrometry data

Raw files obtained from DIA runs were processed using DIA-NN 2.2.0. A FASTA file (UniProt *V. cholerae* serotype O1 reference proteome (taxon ID: 243277) or *E. coli* (strain K12) reference proteome (taxon ID: 83333) was provided. A library-free search was enabled with Trypsin/P protease, two missed cleavages, peptide length range 7–30, precursor charge range 1–4, precursor m/z range 300–1,800, fragment ion m/z range 200–1,800. Cysteine carbamidomethylation was set as a fixed modification. Precursor FDR was set to 1%, and MBR was activated. All other settings were left as default. Using the resulting “report.tsv” file as input, the data were uploaded to DIA Analyst v.0.10.4 (https://analyst-suites.org/apps/dia-analyst/) with 0.05 as an adjusted *P* value cutoff and 1 as the log2 fold change cutoff. Imputation type was set to Perseus type with no normalization. Type of FDR correction was set to Benjamini-Hochberg. The resulting log_2_ fold changes and adjusted *P* values were visualized in volcano-style scatter plots generated in Python (pandas, NumPy, matplotlib, seaborn). For each comparison, the log_2_ fold change (sign inverted for plotting) was plotted against the −log_10_-adjusted *P* value. Proteins exceeding the fold change and significance thresholds were highlighted by color. Features with −log_10_-adjusted *P* values >4, or alternatively the five most significant hits per regulation direction, were annotated using gene names with collision-avoiding label placement.

## SUPPLEMENTAL MATERIAL

Figures S1 and S2 jb.00155-26-s0001.docx Characterization of *V. cholerae* strains and correlation between PepA binding sites and changes in protein abundance.

Table S1 jb.00155-26-s0002.xlsx Results of proteome analysis.

ASM does not own the copyrights to Supplemental Material that may be linked to, or accessed through, an article. The authors have granted ASM a non-exclusive, world-wide license to publish the Supplemental Material files. Please contact the corresponding author directly for reuse.
