# Supplementary material for: The evolution of a Na+-sensitive Vibrio cholerae mutant unmasks the moonlighting aminopeptidase PepA as a regulator of nhaB Na+/H+ antiporter gene expression
Source: J Bacteriol. 2026 Jul 30;208(8):e00155-26. doi: 10.1128/jb.00155-26 (PMC13492116; doi:10.1128/jb.00155-26)
Supplement: Supplementary file 1 [file jb.00155-26-s0001.docx]

**The evolution of a Na^+^-sensitive *Vibrio cholerae* mutant unmasks the moonlighting aminopeptidase PepA as a regulator of**

***nhaB* Na^+^/H^+^ antiporter gene expression**

Sebastian Herdan^1§^, Katharina Kohm^2§^, Robert Warneke^3^, Florian Roth^2^, Nadja Glaser^2^, Lucia Villani^4^, Nele Rüping^2,4^, Lea Dreher^2,4^, Nicolas Görge^1^, Trung Duc Hoang^4^, Emina Schunke^1^, Claudia C. Häse^5^, Juri Rappsilber^3^, Günter Fritz^1^, Fabian M. Commichau^2^, Johannes Gibhardt^4^, Julia Steuber^1^

^1^FG Cellular Microbiology 190 i, Institute for Biology, University of Hohenheim, Stuttgart, Germany

^2^FG Molecular Microbiology 190 h, Institute for Biology, University of Hohenheim, Stuttgart, Germany

^3^FG Bioanalytics, Technical University of Berlin, Germany

^4^FG Microbial Processes of Nitrogen Transformation 190 y, Institute for Biology, University of Hohenheim, Stuttgart, Germany

^5^Carlson College of Veterinary Medicine, Oregon State University, Corvallis, Oregon, USA

^&^The authors should be regarded as joint first authors

*Correspondence to:

Julia Steuber, [julia.steuber@uni-hohenheim.de](mailto:julia.steuber@uni-hohenheim.de)

Johannes Gibhardt, [johannes.gibhardt@uni-hohenheim.de](mailto:johannes.gibhardt@uni-hohenheim.de)

Fabian Commichau, [fabian.commichau@uni-hohenheim.de](mailto:fabian.commichau@uni-hohenheim.de)

**Content**

**Figure S1.** Morphological characterization of *V. cholerae* strains.

**Figure S2.** Correlation between PepA binding sites and changes in protein abundance.

**References**

**Figure S1. Morphological characterization of *V. cholerae* strains.** The strains were grown on LB (pH 6.2, 171 mM NaCl) to exponential phase, harvested and prepared for scanning electron microscopy. **Top panels:** *V. cholerae* O395-N1 (reference strain) and its variant *V. cholerae nqr nhaA* lacking the Na^+^-NQR and the antiporter NhaA. **Bottom panels:** Suppressor mutants S3, S4 and S7 obtained after prolonged cultivation of *V. cholerae nqr nhaA* on LB_Tris_-NaCl (pH 7.8, 150 mM NaCl). All strains were rod shaped and of similar size.

**Figure S2. Overlap between PepA binding sites and changes in protein abundance.**

(**A**) The PepA binding sites in *V. cholerae* O1 biovar El Tor str. N16961 were previously identified by chromatin immunoprecipitation sequencing (ChIP-Seq) [Gibson *et al.*, 2022]. To visualize their distribution across the chromosomes, the PepA binding sites were extracted and mapped onto the the genome of *V. cholerae* O395-N1. The enrichment factors of the binding sites obtained by ChIP-Seq are shown as bars. Red labelled bars indicate PepA binding sites of genes encoding proteins whose abundance was changed only in S3 or S4, or in both suppressors (fold change > 1 or < -1) (p < 0.05). Purple labelled bars indicate PepA binding sites in *fadR*-*nhaB* and *lptF*-*pepA* intergenic regions, and in the *holC* gene that is located downstream of *pepA*. (**B**) Blue bars indicate PepA binding sites of genes encoding proteins whose abundance remained unchanged.

**References**

**Gibson JS, Gebhardt MJ, Santos RERS, Dove SL, Watnick PI** (2022) Sequestration of a dual DNA-binding protein by *Vibrio cholerae* CRP. Proc Natl Acad Sci USA 119: e2210115119.
